# Mechanochemical Properties of Mucoadhesive Tablets Based on PVP/HPβCD Electrospun Nanofibers as Local Delivery of *Polygoni cuspidati* Extract for Treating Oral Infections

**DOI:** 10.3390/ph16040579

**Published:** 2023-04-12

**Authors:** Magdalena Paczkowska-Walendowska, Daria Szymanowska, Judyta Cielecka-Piontek

**Affiliations:** 1 Department of Pharmacognosy, Poznan University of Medical Sciences, Rokietnicka 3, 60-806 Poznan, Poland; jpiontek@ump.edu.pl; 2 Department of Biotechnology and Food Microbiology, Poznan University of Life Sciences, 48 Wojska Polskiego Street, 60-627 Poznan, Poland; daria.szymanowska@up.poznan.pl

**Keywords:** *Polygoni cuspidati*, polydatin, resveratrol, PVP/HPβCD-based electrospun nanofibers, mucoadhesive tablets, periodontitis

## Abstract

This study investigated the ability of PVP/HPβCD-based electrospun nanofibers to enhance the dissolution rate of poorly soluble polydatin and resveratrol, the main active components of *Polygoni cuspidati* extract. To make a solid unit dosage form that would be easier to administer, extract-loaded nanofibers were ground. SEM examination was used to analyze the nanostructure of the fibers, and the results of the cross-section of the tablets showed that they had maintained their fibrous structure. The release of the active compounds (polydatin and resveratrol) in the mucoadhesive tablets was complete and prolonged in time. Additionally, the possibility of staying on the mucosa for a prolonged time has also been proven for both tablets from PVP/HPβCD-based nanofibers and powder. The appropriate physicochemical properties of the tablets, along with the proven antioxidant, anti-inflammatory, and antibacterial properties of *P. cuspidati* extract, highlight the particular benefits of the mucoadhesive formulation for use as a drug delivery system for periodontal diseases.

## 1. Introduction

Gingivitis and periodontitis, which together make up periodontal disease, are frequent oral infections that affect the tissues that support and surround teeth [1]. According to a recent study, periodontal disease affects 47.2% of adults aged 30 years and older, with the prevalence rising to 70.1% in those who are 65 years and older [2]. It is estimated that oral diseases affect nearly 3.5 billion people worldwide [3]. Periodontitis is an insidious disease, that usually begins with plaque formed from food ingredients, substances contained in saliva, and the waste products of bacteria. Over time, minerals build up on it, and tartar is formed. It grows at the gingival margin and descends closer to the root. Over time, it covers the entire surface of the tooth. It is also a good breeding ground for bacteria, that multiply as it grows [4]. Periodontal disease also develops due to an inappropriate body reaction and the existence of inflammation [5]. It has been established that peripheral blood neutrophils, which are thought to be the primary source of reactive oxygen species, are hyperactive in people with periodontitis. According to recent studies, the inflammation of periodontitis is likely to be accompanied by the hyperactivity of neutrophils [6]. Numerous studies have revealed that oxidative stress, both local and systemic, could be influenced by periodontitis [7]. Therefore, it is appropriate to use antioxidants to treat and prevent the onset of the symptoms of periodontitis.

*Polygoni cuspidati rhizoma et radix* is a promising natural product for the treatment of periodontal disease. Epicatechin, resveratrol, and polydatin comprise a significant portion of the active ingredients, contributing to its efficacy [8]. Active compounds determine its antiviral, antibacterial, and anti-inflammatory effects. These, in turn, can be successfully used topically in the oral cavity to treat periodontitis [9].

Despite numerous advantages and wide application, the essential active ingredients present in the plant raw material are characterized by low solubility. While this is a limiting factor for oral bioavailability, it will also determine therapeutic efficacy when administered topically. A great deal of effort goes into developing systems to counter these limitations. One of the most recent pharmaceutical techniques is electrospinning, which, compared to other traditional solid dispersion methods, can produce nanofibers with improved physicochemical properties of active pharmaceutical ingredient particles, and enhanced dissolution. This is due to the enclosed drug becoming more amorphous [10]. Additionally, the porous structure of nanofibers increases the contact surface with the oral fluids and can increase solubility. The development of electrospun nanofibers to enhance the characteristics of resveratrol, one of the polyphenols, a stilbene derivative, has been studied. PVP-based nanofibers improved resveratrol solubility [11,12], while nano-encapsulation with zein, protected resveratrol from the stomach’s unfavorable pH conditions and released it at a controlled rate in the intestinal tract [13]. While increasing the oral bioavailability, rapid dissolution is a significant advantage, considering the topical application, long-term contact of the pharmaceutical form with the affected area is equally important [14].

In our previous work, polyvinylpyrrolidone/(2-hydroxypropyl)-β-cyclodextrin-based (PVP/HPβCD-based) nanofibers, with increased solubility of active compounds, were produced, and the physicochemical characteristics of the material were thoroughly described [15]. Nevertheless, plant material is characterized by many valuable activities, including microbiological activity, which can be used for topical treatment of infections in the oral cavity. For this purpose, it is necessary to develop a formulation that does not immediately dissolve in saliva. Therefore, this study aimed to prepare mucoadhesive tablets containing electrospun nanofibers, as a strategy for treating oral infections. To the best of our knowledge, no scientific reports currently exist suggesting such an administration.

## 2. Results and Discussion

The possibility of using *Polygoni cuspidati* extract in oral cavity infections, including periodontitis, results from its known antioxidant and anti-inflammatory properties. The extracts obtained in this study were also characterized by the appropriate content of active compounds, expressed as total phenolic content (TPC, 38.01 ± 1.17 mg GAE/1 g plant material), antioxidant activity (in the 2,2-diphenyl-1-picrylhydrazyl (DPPH) assay—IC_50_ = 0.13 ± 0.01 mg/mL) and anti-inflammatory activity (expressed as the ability to inhibit the hyaluronidase enzyme—IC_50_ = 4.05 ± 0.18 mg/mL). While these activities have been extensively described [15,16], little information is available about their microbial activity, and therefore such tests were performed first. Bacteria are the principal inhabitants of the mouth, and primarily comprise *Streptococcus mutans* and *Porphyromonas gingivalis* [17]. Significantly, *Streptococcus* (e.g., *S. mutans*) may form biofilms with *Candida*, to play a pathogenic role, including the development of periodontal diseases [18]. This work presents, for the first time, the microbial activity of both the *P. cuspidate* liquid extract, which is a component of nanofibers, and the lyophilized extract, which is a component of tablets, as well as their main active compounds, such as resveratrol and polydatin (Table 1). *P. cuspidati* extracts exhibited activity against all studied bacteria. Resveratrol, one of the main ingredients, has been shown to reduce oral *S. mutans* biofilm formation, leading to a reduction in dental caries [19,20], which may also explain the activity of the entire extract. Microbial growth has been observed for polydatin because, as a glycoside, it is a carbon source for microorganisms.

The unique properties of electrospun nanofibers based on polyvinylpyrrolidone/cyclodextrin have been demonstrated. An increase in the solubility of active compounds found in *Polygoni cuspidati* extract, i.e., resveratrol and polydatin, was shown [15]. Additionally, a broad biological activity of the extract has been demonstrated, in particular antioxidant and anti-inflammatory activity, expressed by inhibiting the activity of the hyaluronidase enzyme. In addition, the high antibacterial activity of the prepared nanofibers was demonstrated. In the nanofibers, one-third of the mass of the system is extract; hence, after recalculation of the microbiological activity results from Table 1, the microbiological activity of the nanofibers is comparable to that of the liquid extract. These are essential properties, indicating the possibility of using the raw material to treat oral cavity infections, including periodontitis [15,16]. Despite the spectacular increase in the solubility of resveratrol and polydatin, the time of the pharmaceutical form remaining at the site of infection is also significant. Some studies have reported on electrospun nanofiber tableting as a new approach in pharmaceutical technology, aiming to create a modified release form [21,22]. Therefore, mucoadhesive tablets based on electrospun nanofibers, were created.

**Figure 1:**
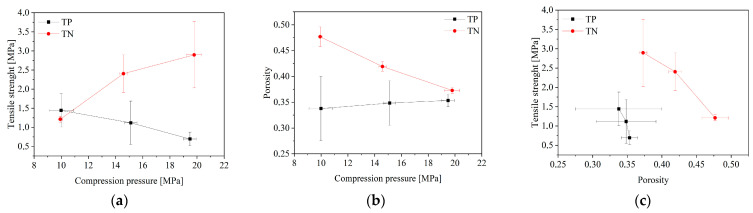
Tabletability (**a**), compressibility (**b**), and compactibility (**c**) profiles of tablets produced from nanofibers (TN) and powder (TP).

The proper amount of PVP/HPβCD-based nanofibers was mixed with magnesium stearate, used as a lubricant (tablets from nanofibers—TN), and corresponding amounts of powder were compressed as control tablets (tablets from powder—TP). Both formulations were tableted correctly and the process was reproducible. Tablet characterization consisted of tabletability, compressibility, and compactibility (Figure 1) [23]. At low compaction pressures, the TN formulation proved its capacity to create the toughest tablets (Figure 1a). Their compressibility was also higher, because a relatively high porosity was retained at higher compression pressure values (Figure 1b). Based on the above-mentioned parameters, the tablets produced from nanofibers have better properties, since they demonstrated a good tabletability (the strongest tablets at the lowest compaction pressures), good compressibility, as well as higher compactibility, compared to the tablets made from powder (the strongest tablet with the highest solid fraction). 

**Figure 2:**
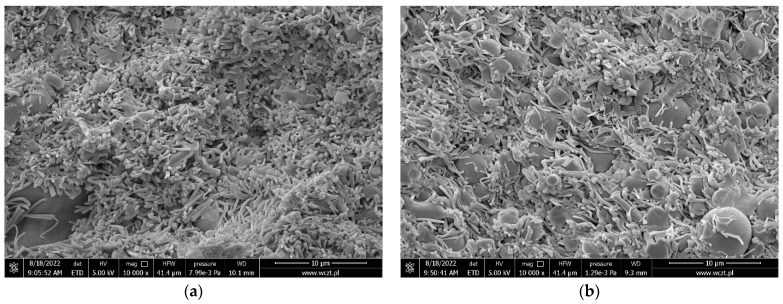

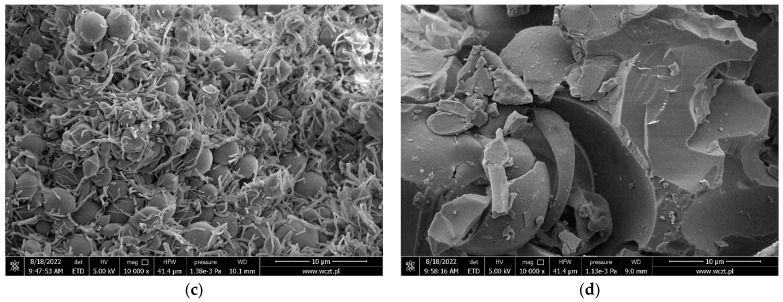
SEM micrographs of the tablets compressed from nanofibers, at a compression force of 500 N (**a**), 750 N (**b**), and 1000 N (**c**), and powder, at a compression force of 750 N (**d**).

For a better understanding of the process underlying the compression of the powder, SEM was used to examine tablet cross-sections at different compression pressures. The mean diameter of the fiber nanostructure was in the nanometer range. The cross-section of the tablet’s fibrous structure was still present, according to SEM, where the tableting process and the applied compression force did not disturb this structure. While in the case of the nanofibers tablets, a decrease in the nanofiber fraction with increasing compression force can be seen (Figure 2a–c). The powder tablets showed severely distorted particles, that looked welded together, likely due to overcompression.

**Figure 3:**
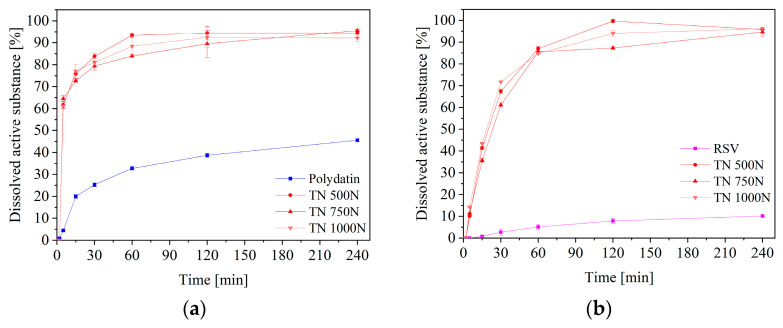

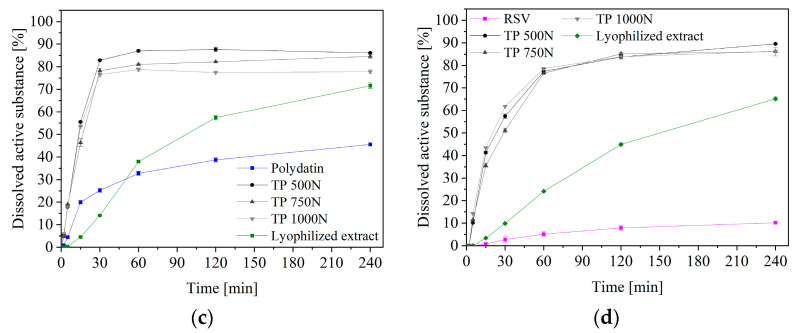
Dissolution profiles of polydatin (**a**) and resveratrol (**b**) from the nanofiber tablets, and polydatin (**c**) and resveratrol (**d**) from the powder tablets, in artificial saliva solution at pH 6.8.

**Figure 4:**
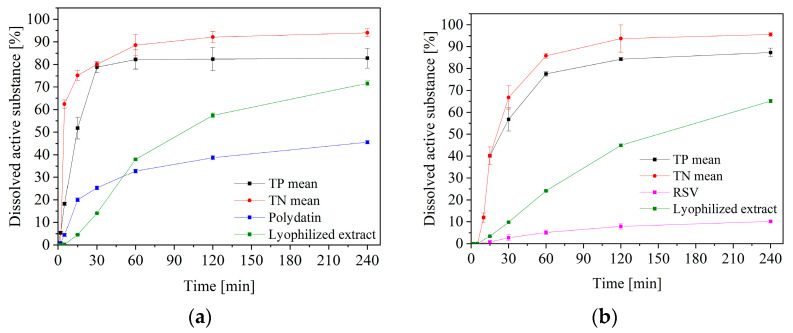
Averaged dissolution profiles of polydatin (**a**) and resveratrol (**b**) from the nanofiber tablets and powder tablets, in artificial saliva solution at pH 6.8.

A very important property from the point of view of the effectiveness of therapy, is the release of active compounds from the formulation. So, the release kinetics of polydatin and resveratrol from both formulations were determined (Figure 3). The dissolution profiles of the TNs compressed at different compression pressures, did not differ statistically (*f*_1_ below 15 and *f*_2_ above 50, close to 100), the same with TPs. However, a statistically significant difference is visible when comparing TN’s with TP’s profiles, as the substances dissolved from the TN reached higher concentrations in the acceptor fluid more quickly (Figure 4). The faster and better release of active substances from tablets based on nanofibers, is due to their greater porosity (Figure 2). Cross-sections of the tablets were analyzed by SEM, and these confirmed the maintenance of the fibrous structure within the tablets. The higher porosity may result in increased water absorption and, consequently, better wettability/dispersibility and more accessible release of the active substance [24]. On the other hand, the visible burst effect, in the case of nanofibers, was slowed down by their compaction, which made it possible for them to remain in an unchanged form longer, which is very important for application in the oral cavity. Similar results were obtained by using PVP-based nanofibers, where the dissolution rate of meloxicam was improved. These results imply that nanofibers could be an effective carrier, to speed up the dissolution of substances [25].

To better illustrate the differences in the release profiles, in the absence of statistically significant differences between the profiles for different compression forces, the profiles for TP and TN were averaged, and are presented in Figure 4.

The dissolution data obtained for both formulations were fitted to the following release models of zero-order and first-order equations, the Higuchi model (applied for the matrix systems), and the Korsmeyer–Peppas model (employed for the swellable matrices), in order to determine the mechanism causing the release of the bioactives (Table 2). The tablets primarily follow the Higuchi model, according to kinetic models that analyzed both formulations. This model suggests that the drug was primarily released through a diffusion mechanism, where the drug release was from the homogeneous planar matrix, which did not disintegrate. To better understand the mechanism of release, it is worth looking at the diffusional exponent n for Korsmeyer–Peppas kinetics, which describes the release as anomalous (for values 0.43 < n < 0.89), suggesting that the combination of diffusion and erosion contributes to the control of drug release [26]. This release mechanism is characteristic of many PVP-based mucoadhesive formulations [27,28].

**Figure 5:**
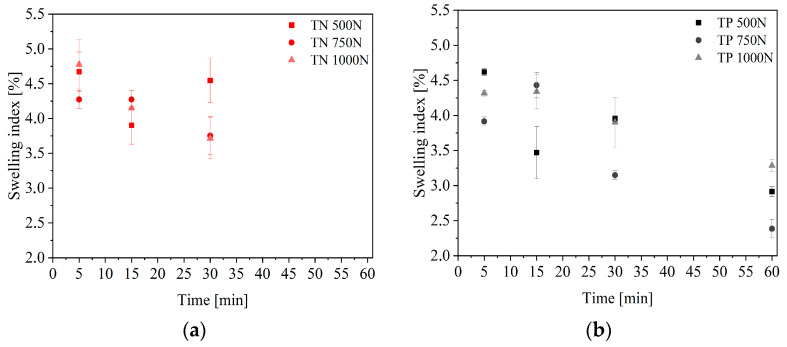
Swelling index of tablets produced from nanofibers (**a**) and powder (**b**).

Because the higher release can be attributed to the higher level of water uptake, which results in enhanced wetting and penetration of water into the film matrices and consequently increased diffusion of the active chemical, swelling studies were conducted, to corroborate the drug delivery mechanism. A matrix’s swelling ratio can be influenced by a number of factors, including hydrophilicity, stiffness, and pore structure. The porous structure of the TN tablets was confirmed, so a higher swelling index for them was also observed (Figure 5). The hydrophilic nature of PVP/CD carriers, may be one of the significant factors that influences the extent of swelling of these matrices [29]. 

Finally, to elaborate on the mucoadhesive behavior of the tablets after prolonged contact with the medium simulating saliva, their residence time was examined (Table 3). All of the examined formulations adhered immediately to the regenerated cellulose membranes and swelled progressively in touch with the acceptor medium throughout the first 25 min, with no apparent indicators of disintegration. The tablets made of PVP/HPβCD-based nanofibers, demonstrated a shorter contact time with the membrane, which may be related to the membrane’s excessive hydration and a quicker matrix disintegration due to the porous nature of the tablets. The weight gain that favored tablet separation from mucosa, and which was greater for the tablets based on nanofibers, was caused by water absorption. Nevertheless, the 30 min contact of the formulation with the membrane, ensures an almost complete release of the active substances (almost 80% for polydatin and 60% for resveratrol), which is a guarantee of the product’s effectiveness.

Finally, biological activity studies were also performed for tableting blends (antioxidant and anti-inflammatory activity tests) or for the final tablets (microbiological tests). The results obtained for the blends/tablets remained at the same level as the results for the starting materials, showing no change in the biological activity of the extract as an active ingredient. Therefore, neither the electrospinning process nor the tableting process had a negative impact on the activity of the entire formulations.

## 3. Materials and Methods

### 3.1. Plant Material

Plant raw material, *Polygonum cuspidatum* rhizome and root, was obtained from Herbapol Cracow (Cracow, Poland) (lot no. 010918).

### 3.2. Chemicals and Reagents

Resveratrol (≥99%, HPLC), polydatin (≥95%, HPLC) were obtained from Sigma-Aldrich (Poznan, Poland). Excipients, such as (2-hydroxypropyl)-β-cyclodextrin (HPβCD), average Mw~1460, and magnesium stearate, were supplied from Sigma-Aldrich (Poznan, Poland), and polyvinylpyrrolidone (PVP) as Kollidon^®^ 30, from BASF Pharma (Burgbernheim, Germany). Reagents for the dissolution studies: potassium chloride, sodium chloride, di-potassium hydrogen orthophosphate, magnesium chloride, calcium chloride and xylitol, and mucoadhesive tests: mucin from porcine stomach, were obtained from Sigma-Aldrich (Poznan, Poland). HPLC grade acetonitrile and water were obtained from Merck. High-quality pure water and ultra-high-quality pure water were prepared using a Direct-Q 3 UV Merck Millipore purification system.

### 3.3. Extract Preparation and Characterization of Phytochemical and Biological Properties

Extract of *Polygoni cuspidati radix* was prepared according to the optimized process described previously [15]. Briefly, the ground plant material was subjected to ultrasound-assisted extraction, using 70% of methanol in the extraction mixture, temperature 70 °C, and four cycles of 20 min. 

Determination of the total phenolic content, the content of selected polyphenols compounds, as well as antioxidant and anti-hyaluronidase activity, have been described previously [15].

Briefly, total phenolic content (TPC) was determined by the Folin–Ciocalteu method. In 96-well plates, 25 µL of the extracts or gallic acid solution (as reference) were added, followed by 200 µL of distilled water, 15 µL of Folin–Ciocalteu reagent, and 60 µL of 20% sodium carbonate solution. To avoid light, the plate was shaken for 5 min at room temperature at 8 rfc (for 600 rpm and rotor radius 2 cm), and then incubated for another 25 min at room temperature. The absorbance was measured at 760 nm. The total content of gallic acid in the produced extracts was estimated using the calibration curve of the standard substance and was presented in milligrams of gallic acid equivalents (GAE) per 1 g of plant material.

Antioxidant activity was measured as a degree of scavenging free DPPH (2,2-diphenyl-1-picrylhydrazyl) radicals. A volume of 175 µL of DPPH solution (3.9 mg/50 mL methanol) was added to 25 µL of extracts, agitated, and then incubated at room temperature for 30 min in the dark. At 517 nm, the absorbance of 25 µL of water, or a 3:7 *v*/*v* mixture of water and methanol and 175 µL of methanol, was measured, in comparison to a blank (25 µL of water or a 3:7 *v*/*v* mixture of water and methanol and 175 µL of methanol). Using the following formula, the percentage of DPPH scavenging activity was calculated:

$$DPPH_{\mathrm{scavenging}\;\mathrm{activity}\;}\left(\%\right)=\frac{A_{0}-A_{1}}{A_{0}}\times 100\%$$

where *A*_0_ is the absorbance of the control, and *A*_1_ is the absorbance of the sample.

The concentration of the extract needed to reduce radical generation by 50%, also expressed as IC_50_ value, was derived from the results.

Anti-inflammatory activity was expressed as inhibition of the hyaluronidase enzyme. The following solutions were combined and incubated for 10 min at 37 °C: 25 µL of enzyme (30 U/mL of acetate buffer pH 7.0), 25 µL of acetate buffer (50 mM, pH 7.0, with 77 mM NaCl, and 1 mg/mL of albumin), 15 µL of acetate buffer (pH 4.5), and 10 µL of extracts. Hyaluronic acid (HA), 25 µL, was then added and incubated for 45 min at 37 °C, with an acetate buffer with pH 4.5. In order to precipitate the undigested HA, 200 µL of 2.5% CTAB in 2% NaOH was added (pH 12). The mixture was left to sit at room temperature for 10 min. The absorbance at 600 nm was used to calculate the turbidity of the reaction mixture. The inhibition percentage was calculated by using the following equation:

$$\%\;inhibition\;activity=\frac{T_{S}-T_{C}}{T_{H}-T_{C}}\times 100\%$$

where *T_S_* is the absorbance of the enzyme + HA + extract, *T_C_* is the absorbance of the enzyme + HA, and *T_H_* is the absorbance of the HA + extract.

The results are expressed as IC_50_ values, which corresponds to the extract concentration required for 50% of hyaluronidase inhibition.

### 3.4. PVP/HPβCD-Based Electrospun Nanofibers Preparation

Electrospun nanofibers were selected in accordance with the DoE methodology described earlier [15]. Briefly, for optimized nanofibers, 2.0 g of *Polygoni cuspidati* extract was combined with 10 mL of ethanol, 2.0 g of HPβCD was added, and the mixture was thoroughly agitated with a magnetic stirrer. Then, 2.0 g of PVP was added, and it was thoroughly mixed with a magnetic stirrer. After being mixed, the solution was poured into a syringe and electrospun at a distance of 12 cm, at a voltage of 25 kV and a flow rate of 2 mL/min. The nanofibers were collected in a rotating collector that was foil-wrapped in aluminum. 

The complete characteristics of the PVP/HPβCD-based nanofibers, their structure and properties, have been described earlier [15] and were not repeated in this work.

#### Microbiological Activity in Liquid Culture

Microorganism strains were created as part of the research’s initial phase. For this, 10 mL of Muller–Hinton liquid propagation medium was used to suspend 0.1 g of bacterial/yeast lyophilisate. To activate and multiply the biomass, the samples were incubated for 18 h, at 37 °C for the bacteria and 30 °C for the yeast. The biomass was centrifuged away from the substrate following incubation (14,000 rpm for 10 min). The pellet was resuspended in 10 mL of 0.9% NaCl and centrifuged once more, after the supernatant was discarded. This procedure was carried out three times. Then, the biomass was diluted in 0.9% NaCl, so that the concentration of microorganisms was 1.0 × 10^2^ cfu/mL. In parallel, a 100 mg/mL solution of each of the tested substances was prepared (the solvent was 0.9% NaCl). The prepared suspension of microorganisms was then added to the dilutions created in this manner, for inoculation. The samples were combined and incubated for 18 h, at 37 °C for the bacteria and 30 °C for the yeast. Using media designed for a specific group of microorganisms, the quantity of bacteria was counted both before and after incubation.

### 3.5. Tableting Process

The flat-faced, 8 mm diameter tablets were compressed using the NP-RD10A tablet press, a single punch, laboratory size tableting device (Natoli, Saint Charles, MO, USA). Utilizing a variety of compaction forces between 500 and 1000 N, the compaction characteristics of the tablets were evaluated (compression pressure in the range of 10 to 20 MPa). The pressure was released after achieving the desired compaction force. Table 4 lists the ingredients of the formulations.

#### 3.5.1. Tablet Characterization

The newly formed tablets were weighed immediately after the compaction. The consistency of the tablet mass was regulated using a method described in Ph.Eur. 9th. Also, the diameter and thickness of 20 randomly selected tablets were measured, using a manual vernier caliper. Following all measurements, mean values and standard deviations (SD) were calculated.

The PTB-M manual tablet hardness testing apparatus was used to measure the tablet hardness, in accordance with the procedures described in Ph.Eur (Natoli, Saint Charles, MO, USA). Each hardness number is the average of six measurements and is expressed as a mean with a standard deviation.

Based on the breaking force (*F*) values (N), the tensile strength (*σ*) values were computed, where *d* is the tablet’s diameter (mm) and *h* is the thickness of the tablet (mm) [30].

$$\;\sigma =\frac{2F}{\pi dh}$$

The following equation, where *W_t_* is the tablet weight (mg), *v* is the tablet volume, and *ρ_true_* is the powder true density (g/cm^3^), was used to calculate the solid fraction (*SF*).

$$\;F=\frac{W_{t}}{\rho_{true}v}$$

The tablet porosity (*ε*) was calculated from *SF* using the following equation:

 ε=1−SF

#### 3.5.2. In Vitro Release Studies

The dissolution investigations were carried out using an Agilent 708-DS dissolving device. A conventional paddle method was used, at 37 ± 0.5 °C, with 0 rfc (for 50 rpm and a 2 cm rotor radius), for stirring. The tablets were dissolved in 300 mL of an artificial saliva solution with the following ingredients: potassium chloride (1.20 g), sodium chloride (0.85 g), di-potassium hydrogen orthophosphate (0.35 g), magnesium chloride (0.05 g), calcium chloride (0.20 g), xylitol (20.0 g) and water up to 1 L; pH was adjusted to 6.8 by 1 M HCl. Liquid samples were taken periodically, and an equivalent volume of a temperature-stabilized medium was substituted. The samples were filtered using a 0.45 mesh nylon membrane filter. The concentrations of polydatin and resveratrol in the filtered acceptor solutions were measured using the previously described HPLC technique [15,16]. The studies preserved sink conditions.

The Moore and Flanner model, which is based on the two-factor values *f*_1_ and *f*_2_, was used to compare the release profiles.

$$f_{1}=\frac{\sum_{j=1}^{n}\left|R_{j}-T_{j}\right|}{\sum_{j=1}^{n}R_{j}}\times 100$$

$$f_{2}=50\times \log\left({\left(1+\left(\frac{1}{n}\right)\sum_{j=1}^{n}{\left|R_{j}-T_{j}\right|}^{2}\right)}^{-\frac{1}{2}}\times 100\right)$$

where *n* is the sampling number, *R_j_* and *T_j_* are the percent dissolved of the reference and test products at each time point *j*. Dissolution profiles are similar when the *f*_1_ value is close to 0 and *f*_2_ is close to 100 (FDA guidelines suggest that two profiles are similar if *f*_2_ is between 50 and 100).

The acquired active compound release patterns were fitted to the following mathematical models to examine the release kinetics: [31]: zero-order equation: F=k×t, first-order equation: nF=k×t, Higuchi equation: $F=kt^{1/2}$, Korsmeyer–Peppas equation: $F=kt^{n}$, where *F* is the fraction of release drug, *k* is the constant associated with the release, and *t* is the time.

#### 3.5.3. Swelling Index

Each tablet was weighed separately and then put into a 25 mL beaker with 10 mL of an artificial saliva solution, at a pH of 6.8 and a temperature of 37 ± 0.5 °C. At predetermined intervals, tablets were removed, cleaned with filter paper, and reweighed (5, 15, 30, and 60 min). The following formula was used to determine the swelling index:

$$\;SI=\frac{W_{2}-W_{1}}{W_{1}}$$

where *SI* is the swelling index, *W*_1_ is the initial weight of the tablet, and *W*_2_ is the weight of the tablet after the particular swelling time interval. 

Each experiment was performed in triplicate.

#### 3.5.4. Determination of the Residence Time

On apparatus modified for the disintegration time test in accordance with prior experiments, the residence time of tablets on the regenerated cellulose membrane simulating swine buccal mucosa, was assessed [16]. In brief, the medium was a pH 6.8 artificial saliva solution, kept at a constant 37 ± 0.5 °C. The foil was brought into contact with each tablet by applying finger pressure for 5 s. Within two hours of the test, the amount of time required to separate the formulation from the mucosal tissue-simulating foil was measured. There were three duplicates of each study.

### 3.6. Statistical Analysis

The Statistica 13.3 software was used to perform the statistical analysis. The Shapiro–Wilk test was used to determine whether the data was normal. The ANOVA test and the post hoc Tukey’s range test for multiple comparisons, were used to analyze the variances between the mean values. Differences across groups were considered significant at *p* < 0.05.

## 4. Conclusions

Mucoadhesive tablets containing PVP/HPβCD-based electrospun nanofibers with *Polygoni cuspidati* extract, have been successfully produced. The release of the active compounds (polydatin and resveratrol) in the prepared tablets was complete and prolonged in time, due to the interactions with the tablet matrix. The proven porous structure of the tablets was a response to the form that facilitates the release of active substances. Additionally, the ability to stay on the mucosa for a prolonged time, has also been proven, for both tablets from nanofibers and powder. So, this work is a proof of a concept, to validate the ability of tableted PVP/HPβCD-based electrospun nanofibers to increase and control the release of active compounds of polydatin and resveratrol from *Polygoni cuspidati* extract. 

In order to ensure patient compliance with small tablet sizes, smooth edges, and low stiffness during treatment, the tablets containing PVP/HPCD-based electrospun nanofibers might be strong enough to be easily inserted close to the periodontal pocket and sustainably release the incorporated bioactives. These qualities point to the various advantages of mucoadhesive formulations for use as a drug delivery mechanism for periodontal diseases, together with the antioxidant, anti-inflammatory, and antibacterial capabilities of *P. cuspidati* extract that have been demonstrated.

## Figures and Tables

**Table 1:** Influence of the material (resveratrol, polydatin, *Polygoni cuspidati* extracts and nanofibers) on indicator microorganisms (reference strains and clinical isolates) by liquid culture method—concentration of the test sample = 100 mg/mL.

	Resveratrol	Polydatin	*P. cuspidate* Liquid Extract	*P. cuspidate * Lyophilized Extract	Nanofibers
	Number of microorganisms [CFU]				
*Escherichia coli* ATCC13706	1.9 × 10^2^ → 3.3 × 10^2^	2.1 × 10^2^ → 3.9 × 10^4^	2.0 × 10^2^ → nd	1.9 × 10^2^ → nd	1.2 × 10^2^ → 3.0 × 10^3^
*Escherichia coli* —clinical isolates	1.4 × 10^2^ → 5.9 × 10^2^	1.3 × 10^2^ → 8.0 × 10^3^	1.9 × 10^2^ → nd	1.4 × 10^2^ → nd	1.9 × 10^2^ → 5.1 × 10^3^
*Pseudomonas aeruginosa* ATCC27853	1.4 × 10^2^ → 3.0 × 10^2^	1.4 × 10^2^ → 4.7 × 10^3^	1.0 × 10^2^ → nd	1.1 × 10^2^ → nd	1.4 × 10^2^ → 3.3 × 10^2^
*Pseudomonas aeruginosa* —clinical isolates	1.1 × 10^2^ → 3.6 × 10^2^	1.8 × 10^2^ → 3.3 × 10^6^	1.0 × 10^2^ → nd	1.5 × 10^2^ → nd	1.1 × 10^2^ → 3.0 × 10^2^
*Streptococcus mutans* ATCC25175	1.2 × 10^2^ → 3.3 × 10^3^	1.0 × 10^2^ → 3.0 × 10^5^	1.0 × 10^2^ → nd	1.9 × 10^2^ → nd	1.3 × 10^2^ → 3.1 × 10^3^
*Streptococcus mutans* —clinical isolates	1.1 × 10^2^ → 1.9 × 10^2^	1.3 × 10^2^ → 8.2 × 10^5^	1.9 × 10^2^ → nd	1.4 × 10^2^ → nd	1.9 × 10^2^ → 5.2 × 10^3^
*Streptococcus salivarius* ATCC 25975	3.4 × 10^2^ → 3.0 × 10^3^	2.9 × 10^2^ → 6.9 × 10^5^	1.9 × 10^2^ → 5.9 × 10^2^	1.6 × 10^2^ → nd	1.3 × 10^2^ → 5.0 × 10^2^
*Streptococcus salivarius* —clinical isolates	1.4 × 10^2^ → 3.7 × 10^2^	1.0 × 10^2^ → 5.0 × 10^5^	1.1 × 10^2^ → 5.9 × 10^2^	1.4 × 10^2^ → nd	1.5 × 10^2^ → 5.1 × 10^2^
*Staphylococcus aureus* ATCC 6538	1.8 × 10^2^ → 3.6 × 10^4^	1.2 × 10^2^ → 5.1 × 10^5^	1.5 × 10^2^ → nd	1.4 × 10^2^ → nd	1.4 × 10^2^ → 3.4 × 10^2^
*Staphylococcus aureus* —clinical isolates	1.0 × 10^2^ → 1.9 × 10^2^	1.3 × 10^2^ → 3.7 × 10^4^	1.9 × 10^2^ → nd	1.1 × 10^2^ → nd	1.7 × 10^2^ → 3.2 × 10^2^
*Staphylococcus epidermidis* NCTC 11047	1.4 × 10^2^ → 3.7 × 10^3^	2.0 × 10^2^ → 9. × 10^6^	1.1 × 10^2^ → nd	1.2 × 10^2^ → nd	1.0 × 10^2^ → 3.0 × 10^3^
*Staphylococcus epidermidis* —clinical isolates	1.8 × 10^2^ → 1.6 × 10^2^	1.0 × 10^2^ → 1.3 × 10^5^	1.5 × 10^2^ → 6.0 × 10^6^	1.1 × 10^2^ → nd	1.4 × 10^2^ → 6.1 × 10^2^
*Enterobacter aerogenes* ATCC 51697	1.0 × 10^2^ → 3.9 × 10^2^	1.4 × 10^2^ → 5.7 × 10^5^	1.9 × 10^2^ → 3.9 × 10^2^	1.4 × 10^2^ → 5.1 × 10^3^	1.3 × 10^2^ → 5.3 × 10^2^
*Enterobacter aerogenes* —clinical isolates	1.4 × 10^2^ → 2.7 × 10^2^	1.7 × 10^2^ → 2.7 × 10^5^	1.1 × 10^2^ → 5.9 × 10^3^	1.4 × 10^2^ → 3.0 × 10^2^	1.0 × 10^2^ → 2.0 × 10^2^
*Candida albicans* ATCC10231	1.8 × 10^2^ → 2.6 × 10^3^	1.8 × 10^2^ → 9.3 × 10^4^	1.2 × 10^2^ → 2.0 × 10^2^	1.1 × 10^2^ → nd	1.2 × 10^2^ → 3.2 × 10^3^
*Candida albicans* —clinical isolates	1.0 × 10^2^ → 2.0 × 10^3^	1.0 × 10^2^ → 1.4 × 10^4^	1.1 × 10^2^ → 1.9 × 10^3^	1.2 × 10^2^ → 3.6 × 10^2^	1.0 × 10^2^ → 3.1 × 10^3^
*Escherichia coli* ATCC13706	1.9 × 10^2^ → 3.3 × 10^2^	2.1 × 10^2^ → 3.9 × 10^4^	2.0 × 10^2^ → nd	1.9 × 10^2^ → nd	1.2 × 10^2^ → 3.0 × 10^3^
*Escherichia coli*— clinical isolates	1.4 × 10^2^ → 5.9 × 10^2^	1.3 × 10^2^ → 8.0 × 10^3^	1.9 × 10^2^ → nd	1.4 × 10^2^ → nd	1.9 × 10^2^ → 5.1 × 10^3^

nd—not detected.

**Table 2:** Mathematical models of release kinetics of polydatin and resveratrol.

	Mathematical Model							
	Zero-Order Kinetics		First-Order Kinetics		Higuchi Kinetics		Korsmeyer–Peppas Kinetics	
	K	R^2^	K	R^2^	K	R^2^	R^2^	n
Polydatin								
Tablets (nanofibers)	16.84	0.36	0.69	0.22	14.12	**0.84**	0.72	0.68
Tablets (powder)	17.47	0.44	0.64	0.29	13.21	**0.89**	0.84	0.63
Resveratrol								
Tablets (nanofibers)	23.00	0.59	0.84	0.36	14.83	**0.93**	0.86	0.71
Tablets (powder)	20.82	0.61	0.82	0.36	13.37	**0.93**	0.85	0.69

Highest scores in bold.

**Table 3:** The residence time of the tablets.

Formulation	TN	TP
Residence time (min)	30 ± 3	50 ± 5

**Table 4:** The composition of tablet formulations.

		Tablets from Nanofibers (TN)	Tablets from Powder (TP)
		Content (mg) per Tablet	
Nanofibers	Extract	33.(3)	-
	HPβCD	33.(3)	-
	PVP	33.(3)	-
Powder	Lyophilised extract (equivalent amount of polydatin and resveratrol)	-	10.0
	HPβCD	-	45.0
	PVP	-	45.0
	Magnesium stearate	1.0	1.0
	Sum	101.0	101.0

## Data Availability

Data is contained within the article.
